# Effects and mechanisms of SGLT2 inhibitors on the NLRP3 inflammasome, with a focus on atherosclerosis

**DOI:** 10.3389/fendo.2022.992937

**Published:** 2022-12-15

**Authors:** Liu Yang, Xuejiao Zhang, Qing Wang

**Affiliations:** Department of Endocrinology, China-Japan Union Hospital of Jilin University, Changchun, China

**Keywords:** atherosclerosis, SGLT2 inhibitor, NLRP3, inflammasome, diabetes mellitus

## Abstract

Atherosclerosis is a lipid-driven chronic inflammatory disease that is widespread in the walls of large and medium-sized arteries. Its pathogenesis is not fully understood. The currently known pathogenesis includes activation of pro-inflammatory signaling pathways in the body, increased oxidative stress, and increased expression of cytokines/chemokines. In the innate immune response, inflammatory vesicles are an important component with the ability to promote the expression and maturation of inflammatory factors, release large amounts of inflammatory cytokines, trigger a cascade of inflammatory responses, and clear pathogens and damaged cells. Studies in the last few years have demonstrated that NLRP3 inflammatory vesicles play a crucial role in the development of atherosclerosis as well as its complications. Several studies have shown that NLRP3 binding to ligands promotes inflammasome formation, activates caspase-1, and ultimately promotes its maturation and the maturation and production of IL-1β and IL-18. IL-1β and IL-18 are considered to be the two most prominent inflammatory cytokines in the inflammasome that promote the development of atherosclerosis. SGLT2 inhibitors are novel hypoglycemic agents that also have significant antiatherosclerotic effects. However, their exact mechanism is not yet clear. This article is a review of the literature on the effects and mechanisms of SGLT2 inhibitors on the NLRP3 inflammasome, focusing on their role in antiatherosclerosis.

## Introduction

Atherosclerosis is a lipid-driven chronic inflammatory disease that is widespread in the walls of large and medium-sized arteries (1, 2). In recent years, fatal vascular diseases caused by atherosclerosis have included stroke, acute myocardial infarction (MI), and severe peripheral vascular disease (3, 4). Pathological changes in atherosclerosis include endothelial damage, lipid deposition, macrophages, the formation of foam cells and the proliferation and migration of smooth muscle cells (SMCs) (5). Previous research has shown that the complex pathogenesis of atherosclerosis includes activation of proinflammatory signaling pathways in the body, increased oxidative stress, and increased expression of cytokines/chemokines (6). Its main causes include lipid accumulation in the arterial wall and chronic inflammation (2, 7, 8). Diabetes has been shown to be an independent risk contributor to the accelerated progression of atherosclerosis (9–11). Research has shown that the rate of vascular disorders in T2DM patients is two to four times higher than that in nondiabetic patients (12). Diabetic patients are prone to atherosclerosis, which is influenced by hyperglycemia, advanced glycosylation end product production, dyslipidemia, inflammation, insulin resistance, endothelial dysfunction and oxidative stress (4, 9, 13).

The results of several recent studies have shown that inflammation is crucial for the onset and progression of atherosclerosis and its complications (1, 14). The existence of inflammasomes was first demonstrated by Martinon et al. in 2002 (15). It is a protein complex that is composed of pattern recognition receptors (PRRs) activated by various physiological or causative stimuli (16). Pattern recognition receptors (PRRs) target pathogenicity in the innate immune response. The inflammasome is an essential part of the innate immune system and has the capacity to promote the expression and maturation of inflammatory factors, release large amounts of inflammatory cytokines, trigger a cascade of inflammatory responses and clear pathogens and damaged cells (17, 18). The most typically characterized and widely studied inflammasomes are the nucleotide binding domain and leucine-rich repeat containing family pyrin domain containing 3 (NLRP3) (19). NLRP3 is an innate immune cell sensor (20). It identifies nonmicrobiological red flags and causes and promotes a bacterial inflammatory reaction in different disease conditions (17, 21, 22). The NLRP3 inflammasome consists of three parts (8, 23–26): (1) The sensor molecule NLRP3 (a toll-like receptor), which includes three structural domains: a pyrin structural domain (PYD), a nucleoside oligomerization structural domain (NACHT) and a leucine-rich repeat sequence structural domain (LRR). (2) Apoptotic spot-like protein (ASC), containing an N-terminal pyrin domain (PYD) and a C-terminal PYD recruitment domain (CARD), also called Pycard. (3) Procaspase-1 contains a CARD and a catalytic structural domain (Caspase-1). ASC plays a critical linkage role between receptor NLRP3 and effector caspase-1 due to the lack of pyrin domain in caspase-1 (27). The NLRP3 inflammasome is a concentric circle-shaped tissue. The NLRP3 protein is located in the middle of the circle, and the ASC layer surrounds the NLRP3 protein. Caspase-1 is located in the outermost layer and is attached to the ASC layer (17, 28). The body can regulate the activation of the NLRP3 inflammasome at multiple levels, and in macrophages, two independent signals are usually required (29). First, inflammatory factors induce NLRP3 and pro-IL-1β expression through stimulation of nuclear factor-κβ (NF-κβ) (30, 31). When pathogen-associated molecular patterns (PAMPs) and damage-associated molecular patterns (DAMPs) are activated, this leads to the interaction of PYD of NLRP3 inflammatory vesicle proteins with PYD of ASC. The accumulation of these structural domains eventually leads to the release of active caspase-1, converting inactive procaspase-1 to active caspase-1 through the action of NLRP3 inflammatory vesicles, thereby upregulating multiple inflammatory cytokines and initiating defense mechanisms (29, 32). Inactive pro-interleukin-1β (Pro-IL-1β) can be converted to active IL-1β by caspase-1 and released from cells to mediate inflammatory responses in tissues (25, 30, 33–35). Active caspase-1 is able to cleave gas protein D (GSDMD), causing the N-terminal structural domain of GSDMD to form a pore in the plasma membrane, which triggers cell lysis until death, also known as pyroptosis (36–39). Pyroptosis is a form of cell death that is distinct from apoptosis. It forces the exposure of intracellular pathogens to other immune factors and triggers the release of cytokines and the production of DAMPs to enhance the immune system’s response to infection (29, 38, 40). Activation of NLRP3 inflammatory vesicles is directly associated with the pathophysiology of chronic inflammatory diseases, such as diabetes, and its associated complications (29, 41–43). Wan et al. found that NLRP3 expression levels and plasma IL-1β levels are dramatically higher in PBMCs from diabetic individuals than in those from healthy controls (44). In addition, aberrant activation of the NLRP3 inflammasome is related to several types of inflammatory diseases, including obesity (45), diabetes (46, 47), atherosclerosis (8, 48, 49), nonalcoholic steatohepatitis (50), gout (51–53), and Alzheimer’s disease (54, 55).

## Current status of research on the NLRP3 inflammasome and atherosclerosis

The NLRP3 inflammasome is mainly expressed in monocytes, macrophages, smooth muscle cells, endothelial cells, and dendritic cells (24, 56, 57). Nevertheless, studies of atherosclerosis have concentrated on the activation of inflammatory vesicles in monocytes, macrophages and vascular smooth muscle cells (24). Vascular smooth muscle cells (VSMCs) are the mesangial cells of coronary arteries (58). In the early stage of atherosclerosis, activated vascular smooth muscle cells have a good ability to proliferate and migrate. These cells can migrate from the arterial mesothelium to the intima, forming fibrous cap-like structures. The fibrous cap secretes extracellular matrix, which buries lipids in deeper layers and prevents plaque degradation. Massive lipid deposition in plaques leads to activation of the NLRP3 inflammatory vesicle response and exacerbation of the inflammatory response, ultimately leading to plaque necrosis (59). It has been shown that in foam cells and macrophages, NLRP3 inflammatory vesicles are mainly localized in the cytoplasm and are associated with intracellular and extracellular crystallization of cholesterol crystals (60). In macrophages, activation of NLRP3 inflammatory vesicles can stimulate the formation of cholesterol crystals, which are necessary for the development of atherosclerosis (49); activation is an important mechanism driving the development of atherosclerotic inflammation (61).

The NLRP3 inflammasome plays an important role in the molecular etiology of atherosclerosis (48, 49, 62, 63). Multiple studies in atherosclerotic patients and animal models have shown that the NLRP3 inflammasome can increase IL-1β and IL-18 production, leading to the progression and instability of atherosclerotic plaques (64). Wan et al. (44) applied NLRP3 knockout technology to inhibit NLRP3 inflammasome activation and the expression of adhesion molecules ICAM-1 and vascular cell adhesion molecule-1 (VCAM-1) in the intima, reducing atherosclerosis and stabilizing atherosclerotic plaques in a diabetic atherosclerosis mouse model. Zheng et al. (65) showed that silencing of the NLRP3 gene delayed the progression of atherosclerosis in mice by administering NLRP3-RNA lentiviral suspension to ApoE-/- mice fed a high-fat diet, mainly by decreasing the plaque content of macrophages and increasing the plaque content of smooth muscle cells. The mRNA levels of NLRP3 inflammasome-related genes are significantly increased in human atherosclerotic plaques compared to nonatherosclerotic vessels, and particularly high expression is observed in patients with symptomatic lesions (66). NLRP3 can be overexpressed in the aorta of patients with atherosclerosis, making it an important risk factor for the development of and correlation with the severity of coronary artery disease (67). NLRP3, ASC, caspase-1, IL-1β, and IL-18 levels are differentially elevated in unstable carotid atherosclerotic plaques in patients undergoing carotid endarterectomy (60, 66, 68). NLRP3 inflammasomes have been shown to be activated by a variety of stimuli, including ion flux, mitochondrial dysfunction, reactive oxygen species production, and lysosomal damage (16, 69, 70). The mechanisms of NLRP3 inflammasome activation are quite complex, and how NLRP3 responds to these signaling events and initiates the assembly of the NLRP3 inflammasome is not fully understood (69). A variety of targets have been used to develop therapeutic strategies. For example, they inhibit the activity of upstream signaling pathways, block the assembly and activation of the NLRP3 inflammasome, inhibit caspase-1 activation and secretion of IL-1 and IL-18 factors (5).

## The role of IL-1β and IL-18 in the pathogenesis of inflammation in atherosclerosis

Several studies have shown that NLRP3 binding to ligands promotes inflammasome formation, activates caspase-1, and ultimately promotes its maturation and the maturation and secretion of IL-1β and IL-18 (66, 71, 72). IL-1β and IL-18 have important roles in the pathogenesis of atherosclerosis (73).

## IL-1β

The interleukin 1 (IL-1) family acts on almost all tissues and cells throughout the body. It plays a key role in innate immunity and inflammatory responses (74). It is a key mediator of inflammatory, autoimmune, infectious and degenerative responses (75). Among all IL-1 family cytokines, IL-1α, IL-1β, IL-18 and IL-1Ra are the most extensively studied members (48). In recent years, interleukin-1β (IL-1β) and interleukin-18 (IL-18) have been identified as the two most important inflammatory cytokines that promote the development of atherosclerosis (76).

IL-1β is known to be a key cytokine in atherogenesis (77, 78). It is an inducible cytokine that is mainly produced by monocytes and macrophages as well as neutrophils (74). IL-1β acts mainly as a soluble mediator outside the cell and can act on tissues and organs at a distance. IL-1β gene expression is low or absent in blood mononuclear cells in healthy populations but significantly increased in disease states (79). Knockout of the IL-1β gene in ApoE-/- mice significantly reduces atherosclerotic plaque development (80). Ablation of the IL-1 receptor (IL-1R) attenuates plaque progression in atherosclerosis-prone mice (81). Active IL-1β has the following main effects: a. Significantly increases the lifespan and activity of neutrophils and macrophages (82) and induces lytic enzymes and fibroblast proliferation (83). b. Induces and controls the expression of genes related to fever, pain threshold, and vasodilation, leading to vascular endothelial cell responses and promoting immune cell responses to infected or injured tissues (84). c. Binding to IL-1R stimulates the activation of the NF-κβ and mitogen-activated protein kinase (MAPK) pathways (85). d. Stimulates the secretion of a range of other cytokines (86) and induces the production of endothelin-1 and adherence molecules in the endothelium, facilitating leukocyte migration and maintaining the cycle of inflammation (87).

## IL-18

A growing number of studies have demonstrated the critical role of IL-18 in atherosclerosis. IL-18 is related to IL-1β both biologically and structurally. Similar to IL-1β, it is generated as an inactive precursor that requires cleavage by caspase-1 to mature into a biologically active cytokine (88). In contrast, unlike IL-1β, IL-18 is constitutively expressed (89). IL-18 binding protein (BP) is an IL-18-specific inhibitor. It is a unique soluble protein that is mainly derived from endothelial cells and monocytes/macrophages. Structurally, IL-18BP has an immunoglobulin (Ig) structural domain (90). IL-18BP binds mature IL-18 (but not pro-IL-18) with high affinity and blocks its interaction with cell surface receptors, thus acting as a natural inhibitor (91). Circulating IL-18BP levels in healthy individuals range from 0.5-7 ng/ml, while elevated IL-18BP levels have been described in a number of autoimmune or inflammatory diseases (92–94). It has been shown that IL-18BP-expressing plasmid DNA prevents the development of fatty streaks in the thoracic aortas of apoE knockout mice and slows the progression of atherosclerotic plaques. IL-18BP has a high binding affinity for IL-18, and IL-18BP is an important regulator of immune and inflammatory responses in IL-18-related diseases (72). In ApoE-/- mouse models, IL-18 has been shown to promote atherogenesis *via* an interferon-γ (IFN-γ)-dependent pathway (95). In contrast, IL-18-deficient ApoE-/- mice show reduced atherosclerotic plaque extension (96). In addition, IL-18 gene variants affect clinical outcomes in patients with coronary artery disease (97). Atherosclerotic lesions are smaller in IL-18 gene-deficient mice (98). Overexpression of IL-18BP and IL-18 deletion in ApoE-/- mice blocked IL-18 activity *in vivo*, leading to impaired development of atherosclerotic injury (48). Studies have shown that IL-18R-/- mice exhibit increased body weight, ectopic lipid deposition, increased inflammation and diminished AMPK signaling pathways in skeletal muscle (99). Elevated IL-18 levels were found in obese and type 2 diabetic patients (100, 101). IL-18 is a costimulatory cytokine that mediates adaptive immunity and is required for interferon-gamma (IFN-γ) production (84).

## The role of an SGLT2 inhibitor on the NLRP3 inflammasome: Possible effects on atherosclerosis

The NLRP3 inflammasome plays an important role in the pathogenesis of atherosclerosis and may therefore be a promising target for therapeutic approaches. Drugs known to date to have targeted inhibitory effects on NLRP3 inflammasomes include statins, metformin (102), colchicine (74), plant compounds (artemisinin, curcumin, rosmarinic acid) (73), and the specific small molecule NLRP3 inhibitor MCC950 (103).

SGLTs are membrane proteins on renal tubular cells whose main role is to transport certain ions and small molecules that mediate glucose reabsorption in the kidney. There are two types of human SGLTs, SGLT1 and SGLT2. SGLT2 is a low-affinity, high-volume cotransporter that is mainly located in the S1 part of the renal proximal tubule and completes approximately 90% of glucose reabsorption. SGLT1 is mainly expressed in the S2 and S3 segments of the brush border of the small intestine and renal proximal tubule and is responsible for glucose transport in the intestinal lumen and reabsorption of the 10% of glucose not reabsorbed by SGLT2 in the renal proximal tubule (104, 105). The site of action of SGLT2 inhibitors is highly specific for the inhibition of renal glucose reabsorption. Sodium-glucose cotransporter-2 inhibitor (SGLT2i) is a blood glucose control drug for the treatment of diabetes. It targets renal glucose reabsorption in an insulin-independent manner to exert its unique hypoglycemic effect (106). The selectivity of SGLT2i for SGLT2 correlated well between *in vivo* and *in vitro* studies (r = 0.985; P < 0.05) (107). SGLT2i regulate glycemic control by enhancing glycosuria, osmotic diuresis and urinary sodium excretion and exert additional positive effects, such as weight loss (108). SGLT2 inhibitors are antiatherosclerotic mainly through several aspects: improving endothelial dysfunction, improving vascular smooth muscle dysfunction, reducing macrophage inflammation and foam cell formation, reducing oxidative stress, reducing inflammation, promoting autophagy, increasing ketone bodies, reducing body weight, lowering blood pressure, and lowering uric acid levels (10, 109–111). Four SGLT2i have been approved for marketing: canagliflozin, dapagliflozin, empagliflozin, and ertugliflozin. A network meta-analysis of randomized controlled trials (RCTs) suggests that engramine may be superior to other SGLT2 inhibitors and has a lower risk of all-cause mortality and cardiovascular events in patients with T2DM (112). However, the main drugs known to have anti-inflammatory effects are dapagliflozin (113) and empagliflozin (114). How SGLT2 inhibitors alter the inflammatory process is not fully understood. Current studies on the effects of SGLT2i on NLRP3 inflammatory vesicles have focused on diabetic nephropathy (17, 115, 116), atherosclerosis (25, 110), steatohepatitis (117), and cardiomyopathy (118).

**Figure 1:**
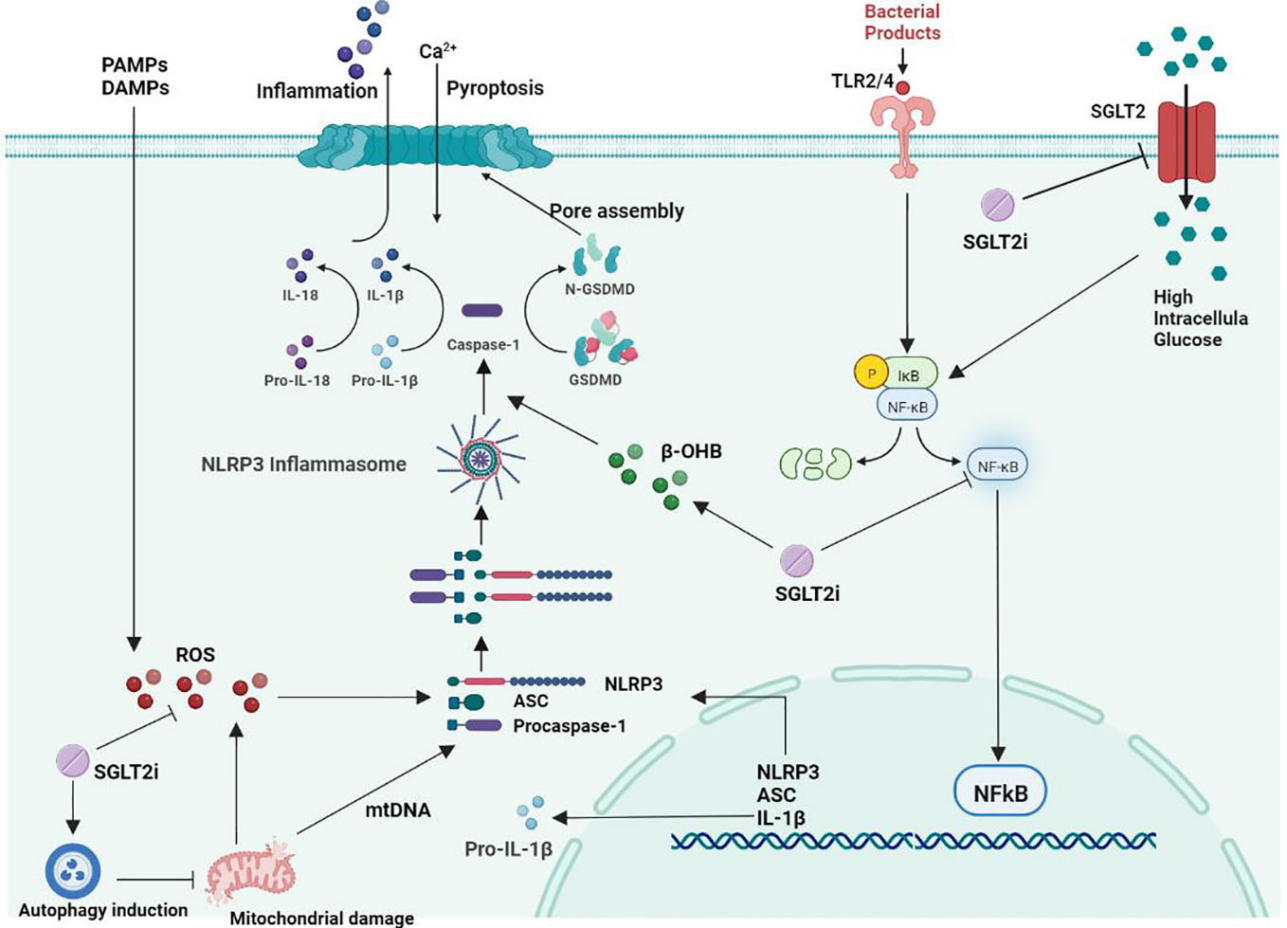
Mechanism of the effect of SGLT2 inhibitors on NLRP3 inflammatory vesicles in atherosclerosis.

A study showed that SGLT2i treatment in diabetic mice improved atherosclerotic plaque regression while lowering blood glucose levels (119). After 30 days of SGLT2i treatment in patients with T2D combined with high cardiovascular risk, NLRP3 inflammasome activity in macrophages was dramatically attenuated, while IL-1β secretion was markedly reduced (120). This may be related to its ability to inhibit atherosclerosis by improving poor glucose tolerance associated with suppression of inflammation (121). SGLT2i can significantly inhibit the formation and development of aortic lesions in diabetic ApoE−/− mice and delay the formation of diabetic atherosclerosis (25). It has been shown that SGLT2 inhibitors can act directly on inflammatory pathways independent of hypoglycemic pathways (111). SGLT2 inhibitors are safe and well tolerated in adults who are overweight and obese but do not have diabetes (122). Under normal glucose conditions, SGLT2i inhibits SMC migration and proliferation by targeting il-17a-mediated oxidative stress, NLRP3 expression and inflammatory responses without inducing cell death (123). Synthesizing the current literature as well as research results, we summarized the effects and mechanisms of SGLT2i on the NLRP3 inflammasome in atherosclerosis treatment (**Figure 1**).

### Direct action mechanisms

#### Reduction of oxidative stress

Oxidative stress is a state of imbalance in the body that favors increased production of reactive oxygen species (ROS) and reduced antioxidant defense systems, resulting in abnormal cell signaling and dysfunction (124). As research progresses, there is increasing evidence that ROS play a more important role in atherogenesis than natural LDL. ROS play an essential role in the inflammatory response, apoptosis, cell growth, alterations in vascular tone, and oxidation of LDL cholesterol (125). Oxidative stress has emerged as a key factor in the pathogenesis of atherosclerosis (6). Studies have demonstrated that ROS generation in the vessel wall is increased in people with risk elements for atherosclerotic cardiovascular disease (CVD) (126). The importance of ROS for macrophage-mediated immune responses is unquestionable (127). Obesity can cause inflammation and insulin resistance, the key cause of which is the recruitment and polarization of macrophages (128). ROS play a key role in NLRP3 inflammasome activation (129, 130), and excessive ROS production is associated with vascular injury. All NLRP3 agonists trigger the production of ROS, which activate NLRP3 inflammatory vesicles through interaction with ROS-sensitive thioredoxin reductase (TXNIP) (25).

DiMarco et al. demonstrated that accelerated atherosclerosis in diabetes is associated with elevated ROS (131). SMC proliferation and migration play a key role in the pathogenesis of atherosclerosis. Persistent inflammation and oxidative stress are also involved in the development of vasoproliferative diseases. In a study by Sukhanov et al., human aortic smooth muscle cells (SMCs) were found to express SGLT2 mRNA and protein, and the application of SGLT2i treatment under normal glucose conditions reduced oxidative stress, NLRP3 expression, SMC migration and proliferation and did not induce cell death (123). Leng et al. showed that SGLT2i reduces the development of atherosclerosis in the aortic root, mainly by lowering blood glucose and lipids to inhibit the ROS-NLRP3-caspaspase-1 pathway in macrophages and reduce IL-1β and IL-18 production (25). In another study, Leng et al. effectively prevented hepatocyte inflammation by treating double HFD/STZ-fed ApoE-/- mice with SGLT2i, suggesting that SGLT2i may be involved in the inhibitory activity of ROS-NLRP3 inflammatory vesicles (117).

#### Inhibition of the NF-κB signaling pathway

Sodium-glucose cotransporter 2 (SGLT2) is a transmembrane protein that transports large amounts of glucose from the extracellular to the intracellular compartment in the diabetic setting. Excess intracellular glucose can induce the activation of NF-kB, ultimately leading to increased expression of the proinflammatory molecule HMGB1 (132). HMGB1 is a DNA-binding protein in the nucleus, and when cells are activated to release HMGB1, it can act as a strong mediator of inflammation by inducing activation of its surface receptors RAGE and TLR-4, thereby increasing the activity of the NF-kB signaling pathway. SGLT2 inhibition may improve ROS, lipid peroxidation and NLRP3-related pathways by attenuating glucose accumulation in renal tubular cells and attenuating HMGB1 and RAGE/TLR-4 expression, thereby reducing NF-κB signaling pathway activity (133–135). Activation of NLRP3 inflammatory vesicles involves multiple signaling pathways, and the pathways interact with each other, of which the NF-κB signaling pathway is an essential part of the NLRP3 activation process (136). In atherosclerotic pathology, NF-κB regulates the expression of several genes, including cytokines (TNF-α, IL-1 and IL-6), monocyte chemotactic proteins and adhesion protein molecules (137). NF-κB is involved in the inflammatory response by binding to NF-κB inhibitor (IκB) retained in the cytoplasm, which leads to the formation of atherosclerotic plaques as well as plaque instability and rupture (138). Activation of NF-κB can induce the production of pro-IL-1β and increase the synthesis of NLRP3 (19). The effect of SGLT2 inhibitors on the NLRP3 inflammasome may be related to its inhibition of the NF-κB signaling pathway. Abdollahi et al. showed that SGLT2 inhibitors can exert direct anti-inflammatory effects independent of glucose concentration, at least in part through inhibition of TLR-4 expression and NF-κB activation and the secretion of proinflammatory mediators (139). Xu et al. showed that SGLT2i ameliorated inflammatory changes induced by NF-κB pathway inhibition in diabetic proximal renal tubular cells with mature IL-1β, IL-6 and TNF-α expression (115). By treating ApoE -/- mice that were induced with atherosclerosis with SGLT2 inhibitors, Liu et al. found that SGLT2 inhibitors significantly reduced inflammation levels *in vivo*, mainly by modulating NF-κB signaling to inhibit IL-1β expression in oxLDL-treated macrophages (140). The above studies suggest that SGLT2 inhibitors can exert their anti-inflammatory effects by inhibiting the NF-κB signaling pathway, and this anti-inflammatory effect is associated with restricted activation of the NLRP3 inflammasome.

#### Activation of cellular autophagy

Autophagy refers to a mechanism by which intracellular macromolecules (such as organelles and protein aggregates) are broken down into their component parts and recycled within the lysosome (141). Defective or dysfunctional components of the cell are degraded. When mitochondrial autophagy removal is impaired, it can lead to reduced release of mitochondria-derived DAMPs and inhibit inflammasome activation (142). The autophagic status also influences the development and progression of atherosclerosis. The autophagy-deficient environment can exacerbate cholesterol crystal-mediated hyperactivation of macrophage inflammasomes and their atherogenic IL-1β response. Previous studies have shown that macrophage autophagy is impaired in atherosclerotic lesions in low-density lipoprotein receptor- or apolipoprotein E-knockout mice (143, 144).

Currently, an increasing number of studies are focusing on the relationship between NLRP3 inflammatory vesicles and autophagy. The mechanism of autophagy inhibition by NLRP3 inflammatory vesicles may lead to ASC reduction, NLRP3 phosphorylation and mitochondrial ROS clearance. Inhibition of cellular autophagy can lead to the accumulation of damaged mitochondria and ROS, which ultimately positively affects NLRP3 inflammasome activity, thereby mediating the inflammatory response (129). Autophagy has been suggested to be the ultimate cellular degradation system of the NLRP3 inflammasome (145). Numerous studies have further shown that autophagy can regulate inflammasome activation through multiple mechanisms, including the NLRP3 inflammasome (146–148). An SGLT2 inhibitor can restore phosphorylated AMPK and autophagy levels induced by high glucose in a dose-dependent manner, thereby inhibiting the activation of the NLRP3 inflammasome (115). Multiple studies have shown that SGLT2 inhibitor-mediated upregulation of autophagy can reduce NLRP3 inflammasome expression, thereby attenuating cardiomyocyte dysfunction and endothelial damage (33, 149).

### Indirect mechanism of action: increase in β-hydroxybutyric acid

Unlike other hypoglycemic agents, specific SGLT2i inhibit glucose reabsorption by proximal renal tubular cells, and the hypoglycemic effect can be independent of pancreatic cell function and insulin sensitivity. This insulin-independent decrease in blood glucose levels reduces the body’s need for insulin and induces an increase in the glucagon-to-insulin ratio (150). Systemic energy metabolism shifts to relative glucose deficiency and triggers increased lipolysis in adipocytes, fatty acid oxidation, and ketone body production in the liver (151, 152). It has been shown that SGLT2i can cause more than doubling of the rate of WAT lipolysis in rats (153), and WAT lipolysis can produce large amounts of β-hydroxybutyric acid, acetoacetic acid and acetone (154). SGLT2 inhibitors stimulate lipolysis and induce mild ketogenic effects in patients with type 2 diabetes (155, 156). β-hydroxybutyric acid (β-OHB) is an endogenous NLRP3 inflammasome inhibitor that can reduce the inflammatory response (157). β-OHB is an NLRP3 inflammasome inhibitor that can reduce NLRP3 inflammatory vesicle-mediated production of IL-1β and IL-18 in human monocytes (158). Bae et al. demonstrated that β-OHB can inhibit endoplasmic reticulum stress and NLRP3 inflammasome activity in rats by activating the AMPK pathway (159). Youm et al. found that β-OHB inhibited NLRP3 inflammasome activation and reduced macrophage and IL-1β production to stop the progression of atherosclerosis (160). However, supplementation, such as ketone supplements, can lead to the acute elevation of β-OHB in the blood, which can significantly increase the activation of caspase-1 and the secretion of the proinflammatory cytokine IL-1β in whole blood (62, 161–163). However, if supplementation with ketone supplements, for example, leads to an acute elevation of β-OHB in the blood, it can significantly increase activation of caspase-1 and increase secretion of the proinflammatory cytokine IL-1β in whole blood (161–163). Multiple studies (120, 164) have shown that in patients with T2D combined with CVD, SGLT2 inhibitors significantly inhibit NLRP3 inflammasome activation and IL-1β secretion in human macrophages by increasing serum β-OHB levels and decreasing serum insulin, glucose, and uric acid levels.

In addition, SGLT2i can affect atherosclerosis as well as cardiovascular events through multiple actions. Tighter glycemic control through SGLT2i treatment can reduce the number of monocytes, improve plasma lipoprotein profiles, and have a significant positive impact on the inhibition of atherosclerosis (165). SGLT2 inhibitors reduce the rate of major adverse cardiovascular events and heart failure hospitalizations in patients with T2DM, regardless of the presence of CVD (166, 167). SGLT2 inhibitors reduce the risk of new ventricular arrhythmic events in patients with T2DM combined with AMI (168). DAPA reduces intima-media thickening, eliminates cardiac hypertrophy and myocardial injury, reduces cardiac inflammation and fibrosis (169), and has an effect on myocardial remodeling (170). In addition, engramine may reduce debilitating conditions in patients with diabetes and hypertension (171). During treatment with SGLT2 inhibitors, a beneficial effect on the circadian rhythm of BP and sympathetic nerve activity (SNA) was demonstrated, resulting in a decrease in BP without a concomitant compensatory increase in HR (172). Treatment with SGLT2 inhibitors in T2DM patients treated with coronary artery bypass grafting (CABG) significantly reduced the amount of inflammatory factors such as IL-1, IL-6 and TNF-α (173).

## Conclusion

In summary, NLPR3 inflammasome production has an important role in promoting the development of atherosclerosis. SGLT2 inhibitors can inhibit the development of atherosclerotic plaques by inhibiting NLRP3 inflammatory vesicles through multiple pathways. However, there may be crosstalk between the above mechanisms; for example, increased intracellular ROS levels can promote the activation of the NF-κB signaling pathway and the initiation of cellular autophagy, cellular autophagy can reduce intracellular ROS levels and inhibit the activation of the NF-κB signaling pathway, and β-OHB can promote myocardial autophagic flux and reduce the formation of mitochondrial ROS. A variety of SGLT2i have been shown to attenuate atherosclerotic lesions in animal models of diabetes (25, 174–176), and overall trial data estimated a relative reduction in the incidence of MACE (HR 0.89, 95% CI 0.82-0.96) and stroke (HR 0.92, 95% CI 0.79-1.08) of approximately 11% (177). In diabetic ApoE knockout (ApoE-/-) mice, SGLT2i (1 mg/kg/day) increased the aortic root atherosclerotic lesion area by 33% and reduced the atherosclerotic plaque size by 27% (178). Drugs specific for the treatment of atherosclerosis are still under further investigation. Indeed, the potential mechanism of SGLT2 inhibitors on the CV system is unclear (110). The results of the EMPA-REG study (179) suggest that SGLT2i activate a nonclassical RAAS pathway, namely, the angiotensin II type 2 receptor. Activation of angiotensin II type 2 receptors protects the cardiovascular system through multiple mechanisms, mainly including vasodilation, increased sodium excretion, anti-inflammation, and anti-arrhythmia. It has also been shown that SGLT2i may reduce cardiovascular (CV) risk in patients with type 2 diabetes mellitus (T2DM) by affecting the aldosterone/renin ratio (ARR) through diuretic and sympathetic depressant effects (104). An SGLT2 inhibitor, as a hypoglycemic agent, also inhibits atherosclerosis, but its mechanism and therapeutic effect still need more basic research and clinical observation to clarify. What is certain is that patients with type 2 diabetes mellitus combined with atherosclerosis benefit more from SGLT2 inhibitors.

## Author contributions

LY wrote the first draft of the manuscript; LY and XZ for the writing, review and editing; QW revised the manuscript. All authors contributed to the article and approved the submitted version.
